# Burst Fracture of C5 with Traumatic Anterior Spondyloptosis of C6 and Posterior Spondylolisthesis of C4 Vertebra: A Case Report

**DOI:** 10.31729/jnma.5289

**Published:** 2021-04-30

**Authors:** Poojan Kumar Rokaya, Nilam Kumar Khadka, Praveen Kumar Giri, Robin Khapung, Nirajan Mahaseth

**Affiliations:** 1 Department of Orthopedics & Trauma Surgery, Karnali Academy of Health Sciences, Nepal; 2 Department of Neurosurgery, National Academy of Medical Sciences, Nepal; 3 Department of Anaesthesia and Critical Care, Karnali Academy of Health Sciences, Nepal

**Keywords:** *cervical vertebrae*, *injury*, *spondylolisthesis*

## Abstract

Burst fracture of C5 with traumatic anterior spondyloptosis of C6 and posterior spondylolisthesis of C4 vertebra is an exceedingly rare high energy injury. Treatment includes decompression, reduction, stabilization, and fusion via anterior or posterior or combined anterior-posterior approach with or without prior traction. We report this rare subaxial cervical spine injury associated with quadriplegia managed with combined anterior and posterior instrumented fusion. A multidisciplinary approach with preoperative assessment and planning is crucial in managing cervical spine injury. Immediate postoperative critical care support, rehabilitation, and dedicated nursing care are required for a favorable outcome in traumatic quadriplegia.

## INTRODUCTION

Spondyloptosis is the complete slippage of the cranial vertebra concerning the caudal vertebra.^1^ Anterior traumatic spondyloptosis of subaxial cervical spine is rare.^2^ Similarly, posterior spondyloptosis of the cervical spine is very unusual and sparsely reported in the literature.^3^ Common level of injury for traumatic subaxial cervical spondyloptosis is C7-T1.^4–5^ Burst fracture of C5 with traumatic anterior spondyloptosis of C6 and posterior spondylolisthesis of C4 vertebra is exceedingly rare high energy injury. Management of cervical spine injury should be individualized depending upon cord compromise, fracture instability, and posterior ligamentous complex injury. There is persistent debate regarding standard management of cervical spine injuries which comprises conservative care, anterior fusion, posterior fusion, and circumferential fusion.^6^ The preferred method should restore spinal alignment, stability and allow early rehabilitation. We report a case of a burst fracture of C5 with traumatic anterior spondyloptosis of C6 and posterior traumatic spondylolisthesis of C4 vertebra managed with combined anterior and posterior instrumented fusion.

## CASE REPORT

**Figure 1.**
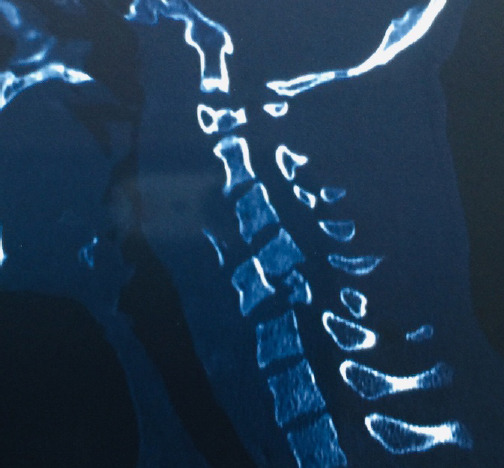
Computed tomography sagittal section showing burst fracture of C5 with spondylolisthesis of C4 and spondyloptosis of C6.

A 22 years old male in remote Nepal was cutting a tree for his agricultural livelihood. He got stuck with the same tree over his neck. He was brought to the emergency with the complaint of neck pain and unable to move his bilateral upper and lower limbs. The cervical spine was protected with Philadelphia hard cervical collar. Glasgow coma scale was 15/15. The neurological level was C3 with a complete injury. Oxygen was given at 6L/min and mean arterial pressure was maintained above 85 mm of Hg with injection noradrenalin. Log roll and back care were advised every two hourly. Chest physiotherapy and nebulization were started. Bowel and bladder care were provided. Plain radiograph and computerized tomography of the cervical spine revealed a fracture of anterior body of C4 vertebra, burst fracture of C5 with retropulsion fragment, fracture of the left lamina and left pedicle of C5, fracture of the bilateral pedicle of C6, anterior superior migration of C6 at the level of C4 and posterior inferior migration of C4 (Figure 1).

Bilateral vertebral arteries were normal on a computerized tomography angiogram. Magnetic resonance imaging is unavailable at our hospital. Gardner wells tong traction was applied in the operating room with a gradual increase in traction weight up to 10 kg to realign the fracture. Preoperative discussion, assessment, and planning were done between the emergency physician, orthopedic surgeon, neurosurgeon, radiologist, and critical care team.

**Figure 2.**
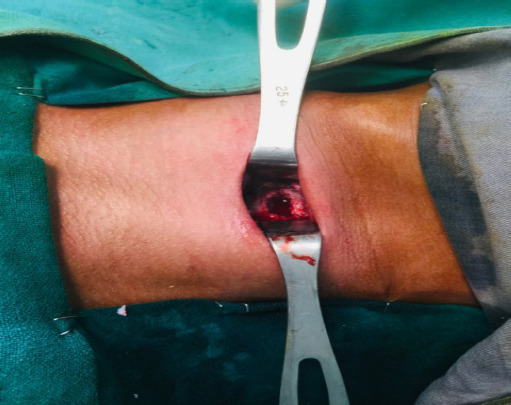
Anterior corpectomy of C5 vertebra.

Initially, in the supine position, we followed Smith and Robinson approach via the longitudinal incision.^6^ Esophagus, trachea, and carotid sheath were gently retracted and protected. Anterior C5 median corpectomy and fracture fragment of C4 were removed with the aid of a microscope and fluoroscopy (Figure 2).

**Figure 3.**
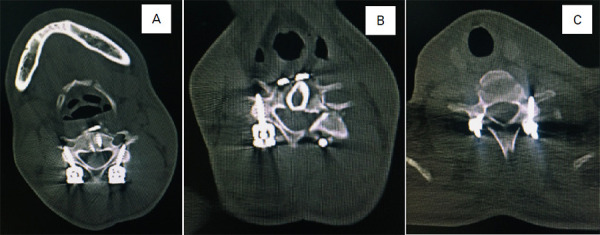
Postoperative computed tomographic axial section of C4 with lateral mass screws (A), C5 right lateral mass screw, fracture of left lamina and pedicle of C5, and autologous fibular strut graft at C5 corpectomy site (B), C7 left lateral mass screw, and right pedicle screw (C).

Posteriorly, a midline incision was used to separate ligament nuchae and paraspinal muscles. Subperiosteal dissection of the spinous process, spinolaminar junction, lamina, lateral mass, and facet joint of bilateral C4C5C6C7 was carried out. Bilateral C4C5, C5C6, C6C7 facet joint was decorticated. Stabilization was done with lateral mass screw over bilateral C4 lateral mass, C5 right lateral mass, and C7 left lateral mass under fluoroscopic guidance (Figure 3).

A pedicle screw was inserted on C7 right pedicle. Connecting rods fixed to lateral mass screw and pedicle screw. The cancellous bone of the C5 vertebra was placed over decorticated spinous process, lamina, lateral mass, and facet joints for posterolateral fusion.

**Figure 4.**
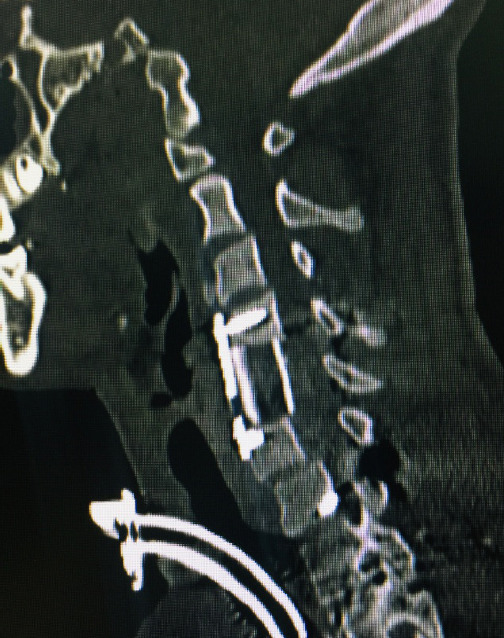
One month postoperative computed tomography sagittal section showing anterior cervical plate and screws at C4 and C6 with fibular graft in between.

An autologous strut graft was harvested from the left mid fibula. Again in the supine position, the anterior column was reconstructed with appropriate size autologous fibular strut graft followed by stabilization with anterior cervical plate and screws (Figure 4).

The patient tolerated the surgery and was shifted to the intensive care unit with ventilator support due to poor respiratory effort. He required inotropes during intensive care unit stay to maintain blood pressure and heart rate.

Tracheostomy was performed on eight postoperative days. At one-month of post-operative time patient is hemodynamically stable with oxygen supplementation through a metallic tracheostomy tube. He has developed a grade II bedsore and bedside mobilization has been started in a wheelchair.

## DISCUSSION

Traumatic spondylolisthesis of subaxial cervical spine at two levels with associated burst fracture is an uncommon injury that poses special challenges to the treating surgeon. Flexion injury over cervical spine from a falling tree led to a burst fracture of C5 with anterior spondyloptosis of C6 and posterior spondylolisthesis of C4 vertebra which is exceedingly rare. Severe canal compromise and posterior ligamentous complex injury could be expected in this high-energy trauma. We decided on global fixation due to the inherently unstable nature of fracture and spondylolisthesis at two levels. There is a lack of common consensus in the literature regarding the standard approach for fixation in cervical spine injuries.^6^ Treatment includes decompression, reduction, stabilization, and fusion via anterior or posterior or combined anteriorposterior approach with or without prior traction.^7,8^ Decision for approach depends upon disc herniation, fracture instability, posterior ligamentous complex injury, surgeons experience, and available resources. Our decision to stabilize from both anterior and posterior side was justified as we noted disrupted posterior ligamentous complex at C4C5 and C5C6 intraoperatively. The preferred surgical approach should allow adequate spinal decompression, restoration of spinal alignment, spinal stability with minimal complications, and facilitate prompt rehabilitation. A multidisciplinary approach with preoperative planning among involved expertise is vital for a favorable postoperative outcome. Immediate postoperative critical care support followed by rehabilitation and dedicated nursing care is important in managing cervical spine injury with quadriplegia.

## References

[1] Akay KM, Ersahin Y, Tabur E (2002). Cervical spondyloptosis: a case report. Minim Invasive Neurosurg.

[2] Munakomi S, Bhattarai B, Cherian I (2015). Traumatic Cervical Spondyloptosis in a Neurologically Stable Patient: A Therapeutic Challenge. Case Rep Crit Care.

[3] Fattahi A, Tabibkhooei A (2019). Traumatic cervical posterior spondyloptosis: report of a rare case. Br J Neurosurg.

[4] Tumialán LM, Dadashev V, Laborde DV, Gupta SK (2009). Management of traumatic cervical spondyloptosis in a neurologically intact patient: case report. Spine (Phila Pa 1976).

[5] Acikbas C, Gurkanlar D (2010). Post-traumatic C7-T1 Spondyloptosis in a patient without neurological deficit: a case report. Turk Neurosurg.

[6] Lee JY, Nassr A, Eck JC, Vaccaro AR (2009). Controversies in the treatment of cervical spine dislocations. Spine J.

[7] Modi JV, Soman SM, Dalal S (2016). Traumatic Cervical Spondyloptosis of the Subaxial Cervical Spine: A Case Series with a Literature Review and a New Classification. Asian Spine J.

[8] Dahdaleh NS, Dlouhy BJ, Greenlee JD, Smoker WR, Hitchon PW (2013). An algorithm for the management of posttraumatic cervical spondyloptosis. J Clin Neurosci.

